# Co-creating a Virtual Reality Program for Older Adults with Dementia in Hospital

**DOI:** 10.1093/geroni/igaf122.3480

**Published:** 2025-12-31

**Authors:** Lillian Hung, Lily Haopu Ren, W Ben Mortenson, Angelica Lim, Jim Mann, Lily Wong, Christine Wallsworth, Jennifer Boger

**Affiliations:** University of British Columbia, Vancouver, British Columbia, Canada; The University of British Columbia, Vancouver, British Columbia, Canada; The University of British Columbia, Vancouver, British Columbia, Canada; Simon Fraser University, Vancouver, British Columbia, Canada; The University of British Columbia, Vancouver, British Columbia, Canada; The University of British Columbia, Vancouver, British Columbia, Canada; The University of British Columbia, Vancouver, British Columbia, Canada; University of Waterloo, Waterloo, Ontario, Canada

## Abstract

Virtual Reality (VR) is promising in improving the well-being of older adults with dementia in hospitals; however, traditional VR design and VR experience delivery to meet their diverse needs. Involving older adult patient partners, family caregivers, and staff in the co-design of gerontechnology is considered best practice, yet few researchers have adopted this inclusive approach. Appreciative Inquiry offers a collaborative framework to engage relevant users, leveraging their expertise and lived experiences for innovation. Our study aims to understand the contribution of Appreciative Inquiry in engaging multiple partners in co-creating a VR program for Older Adults with Dementia in hospitals. We co-created a VR program guided by Appreciative Inquiry principles, engaging patient partners, families, hospital staff and leaders. We adapted methods to facilitate meaningful participation, including six focus groups and interviews with 56 participants across two hospital units. Participants’ insights informed the iterative development and refinement of the VR program. Patient partners, families, staff, and leaders meaningfully contributed to the design process. The collaborative process fostered a sense of ownership among participants, challenged assumptions about dementia care, and promoted positive interactions and experiences within the project team. Our approach demonstrated that using tailored methods is essential for authentic engagement, ensuring the developed technology meets real-world needs and will more likely be adopted in care practice. The study demonstrates the value of Appreciative Inquiry-informed co-design partnerships in co-creating technology. Future studies should further explore co-design methods across diverse cultural and organizational contexts with diverse older adults and other partners.

